# Curcumin induces apoptosis in JAK2‐mutated cells by the inhibition of JAK2/STAT and mTORC1 pathways

**DOI:** 10.1111/jcmm.14326

**Published:** 2019-04-29

**Authors:** Jessica Petiti, Valentina Rosso, Marco Lo Iacono, Cristina Panuzzo, Chiara Calabrese, Elisabetta Signorino, Lucrezia Pironi, Antonio Cartellà, Enrico Bracco, Barbara Pergolizzi, Tiziana Beltramo, Carmen Fava, Daniela Cilloni

**Affiliations:** ^1^ Department of Clinical and Biological Sciences University of Turin Turin Italy; ^2^ Department of Oncology University of Turin Turin Italy; ^3^ SSD Transfusional Center San Luigi Gonzaga Hospital Turin Italy

**Keywords:** curcumin, JAK/STAT, JAK2 V617F, mTORC1, Myeloproliferative neoplasms

## Abstract

Myeloproliferative neoplasms are chronic myeloid cancers divided in Philadelphia positive and negative. The *JAK2 V617F* is the most common mutation in Philadelphia negative patients and results in a constitutive activation of the JAK/STAT pathway, conferring a proliferative advantage and apoptosis inhibition. Recent studies identified a functional crosstalk between the JAK/STAT and mTOR pathways. The identification of an effective therapy is often difficult, so the availability of new therapeutic approaches might be attractive. Previous studies showed that curcumin, the active principle of the *Curcuma longa*, can suppress JAK2/STAT pathways in different type of cancer and injuries. In this study, we investigated the anti‐proliferative and pro‐apoptotic effects of curcumin in *JAK2 V617F‐*mutated cells. HEL cell line and cells from patients *JAK2 V617F* mutated have been incubated with increasing concentrations of curcumin for different time. Apoptosis and proliferation were evaluated. Subsequently, JAK2/STAT and AKT/mTOR pathways were investigated at both RNA and protein levels. We found that curcumin induces apoptosis and inhibition of proliferation in HEL cells. Furthermore, we showed that curcumin inhibits JAK2/STAT and mTORC1 pathways in *JAK2 V617F‐*mutated cells. This inhibition suggests that curcumin could represent an alternative strategy to be explored for the treatment of patients with myeloproliferative neoplasms.

## 1 INTRODUCTION

Myeloproliferative neoplasms (MPNs) are clonal hematopoietic stem cell disorders characterized by an uncontrolled proliferation of one or more elements of the myeloid lineage.^1^ In addition to chronic myeloid leucemia (CML), characterized by the presence of the Philadelphia chromosome, the Philadelphia negative MPNs are essential thrombocythemia (ET), polycythemia vera (PV) and primary myelofibrosis (PMF). The *JAK2 V617F* is the most frequent mutation in patients affected by PV, ET and PMF. *JAK2* gene encodes for a non‐receptor tyrosine kinase crucial for signal transduction downstream of the erythropoietin, thrombopoetin and related receptors that control erythrocyte and megakaryocyte expansion.^2^ Activated JAK2 phosphorylates STATs proteins, specifically STAT5 and STAT3, that homodimerize and translocate to the nucleus. Activated STATs induce the expression of target genes, such as PIM‐1, PIM‐2 and PIM‐3, serine/threonine kinases that promote cells survival, proliferation and therapy resistance.^3^ *JAK2* mutations directly activate JAK/STAT signalling and make myeloproliferation cytokine independent or hypersensitive. JAK/STAT deregulation is critical for MPNs developing and progression. Furthermore, recent studies identified the role of mTOR pathway in MPNs, highlighting a functional crosstalk between the JAK/STAT and mTOR.^4^, ^5^ mTOR is a serine/threonine kinase that regulates cellular metabolism, growth and survival and it may form different proteins complexes: mTORC1 and mTORC2. mTORC1 is composed of mTOR, Raptor, GβL and DEPTOR and it is regulated by AKT. In normal cells, mTORC1 is essential for erythroid and megakaryocytic differentiation through the activation of downstream effectors including 4eBP1 and p70s6K.^6^ This pathway has been found deregulated particularly in megakaryocytes of MPNs patients.^7^ The deregulation of JAK/STAT and mTOR pathways induces an inflammatory state with aberrant cytokine expression.^8^ Given the heterogeneous clinical needs of MPNs patients, determination of a standard therapeutic protocol is often difficult. Moreover, targeted therapy with JAK inhibitors revealed to have some limits in terms of efficacy,^9^ therefore it is necessary to find additional approaches to improve the results so far obtained.

Curcumin is the active phytochemical component isolated from the rhizome of the *Curcuma longa* plant. Curcumin is a highly pleiotropic molecule with multiple pharmacological effects, such as anti‐inflammatory, anti‐microbial, anti‐oxidative and anti‐proliferative activities.^10^, ^11^ Extensive preclinical trials have indicated curcumin therapeutic potential against a wide range of human diseases.^12^ Previous studies showed that curcumin can suppress JAK2/STAT signalling pathways in different type of cancer and injuries.^13^, ^14^ Chen et al demonstrated that curcumin increased the transcript levels of SOCS‐3, an important negative regulator of JAK2, and significantly inhibited the clonogenic activity of hematopoietic progenitors from MPNs patients.^15^ Furthermore, curcumin was able to dissociate Raptor from mTOR, by inhibiting mTORC1 signalling and the phosphorylation of its downstream effectors in different cell lines.^16^ In this study, we investigated the effect of curcumin on JAK2 V617F cell line and in primary cells from MPNs patients. Our results suggest that curcumin inhibits proliferation and activates cell death program by modulating JAK2/STAT and mTORC1 pathways.

## 2 MATERIALS AND METHODS

### 2.1 Cells culture conditions

HEL cell line was purchased from American Type Culture Collection (ATCC, Manassas, USA). HEL cells were grown in RPMI 1640 medium supplemented with 200 nmol/L Glutamine (EuroClone), 10% inactivated foetal bovine serum (FBS, Sigma‐Aldrich) and 0.1% penicillin/streptomycin and maintained at 37°C with 5% CO_2_.

### 2.2 Patients cohort

After informed consent, human peripheral blood (PB) leucocytes were isolated by Buffy Coat procedure from 30 MPNs patients (24 were PV, 4 ET and 2 PMF; the median age was 63 years (range 20‐86); 18 were males and 12 females) and 10 healthy donors. All samples obtained from patients were *JAK2 V617F* mutated. The study was approved by the ethic committee on 16 December 2015 (number of approval 212/2015).

### 2.3 Cells treatment

HEL cells were incubated with different concentrations (10, 15, 20, 30 µmol/L) of curcumin (stock solution 50 mmol/L in DMSO, #C1386, Sigma‐Aldrich) for 24 and 48 hours. Leucocytes isolated from MPNs patients were incubated with 30 µmol/L of curcumin in IMDM (EuroClone) supplemented with 20% inactivated FBS for 20 hours. After incubation, apoptosis and proliferation were evaluated and total RNA and proteins were extracted as described below.

### 2.4 Apoptosis and viability assays

Apoptosis was evaluated using APC Annexin V (BioLegend), according to the manufacturer's instructions. Cells were analysed by flow cytometry (FACS) and the apoptotic fraction was defined as annexin V positive. HEL cells were treated with increasing concentration of curcumin for 24 and 48 hours. After incubation, cells were harvested, resuspended in Flow Cytometry Staining Buffer (#FC001, R&D Systems) and labelled with 1 μg/μL propidium iodide (PI). Live cells (PI negative fraction) were counted by FACS.

### 2.5 Protein extraction and immunoblotting

To perform immunoblotting analysis, we used human HEL cells and cells from MPNs patients. To isolate total protein content, samples were lysed in ice with Ripa buffer (50 mmol/L Tris‐HCl pH 8.0, 150 mmol/L NaCl, 1% Np40, 0.5% DOC, 0.1% SDS with freshly added of protease and phosphatase inhibitors cocktail [Sigma‐Aldrich]) and cell debris were removed by centrifugation at 14 000*g* at 4°C for 15 minutes. Protein concentration was determined by Bio‐Rad Protein Assay (Bio‐Rad): 30 µg of each total cell lysate were loaded, resolved through SDS‐PAGE 6%‐12% gel and electroblotted onto 0.2 µm nitrocellulose membranes (Bio‐Rad). After blocking with 5% BSA (Sigma‐Aldrich) in TBS 1x plus 0.2% Tween‐20 (Sigma‐Aldrich) for at least 1 hour at RT, membranes were incubated overnight (ON) at 4°C with the primary antibodies listed in Table 1 with a dilution of 1:1000. As a secondary antibody, we used goat antimouse IgG‐HRP (sc‐2005), mouse anti‐goat IgG‐HRP (sc‐2354) and goat anti‐rabbit IgG‐HRP (sc2004) [Santa Cruz Biotechnology] in a dilution of 1:7000 for 1 hour at RT. Immunoreactive bands were visualized by Clarity Western ECL Substrate (Bio‐Rad). Quantification was performed using Image Lab program (Bio‐Rad).

**Table 1:** List of primary antibodies

Antibody	Catalog n°	Company	Species
JAK2 (C‐14)	sc‐34479	Santa Cruz Biotechnology	Goat
p‐JAK2 (Tyr221)	sc‐101718	Santa Cruz Biotechnology	Rabbit
Stat3 (F‐2)	sc‐8019	Santa Cruz Biotechnology	Mouse
Stat5 (A‐9)	sc‐74442	Santa Cruz Biotechnology	Mouse
p‐Akt1/2/3 (Thr308)‐R	sc‐16646‐R	Santa Cruz Biotechnology	Rabbit
Raptor (10E10)	sc‐81537	Santa Cruz Biotechnology	Mouse
GAPDH (A‐3)	Sc‐137179	Santa Cruz Biotechnology	Mouse
p70 S6 kinase α (H‐9)	sc‐8418	Santa Cruz Biotechnology	Mouse
SOCS‐1 (H93)	sc‐9021	Santa Cruz Biotechnology	Rabbit
SOCS‐3 (SO1)	sc‐51699	Santa Cruz Biotechnology	Mouse
Phospho‐ p70 S6 kinase (Thr389)	9208	Cell Signaling Technology	Rabbit
Phospho‐Raptor (Ser792)	2083	Cell Signaling Technology	Rabbit
PDK1	3062	Cell Signaling Technology	Rabbit
Phospho‐PDK1 (Tyr373/376)	3065	Cell Signaling Technology	Rabbit
Phospho‐STAT5 (Tyr694) (D47E7)	4322	Cell Signaling Technology	Rabbit
Phospho‐STAT3 Tyr 705 (D3A7)	9145	Cell Signaling Technology	Rabbit
Akt (pan) (C67E7)	4691	Cell Signaling Technology	Rabbit
4E‐BP1	9452	Cell Signaling Technology	Rabbit
p4E‐BP1	9451	Cell Signaling Technology	Rabbit
Cleaved Caspase‐3	9661	Cell Signaling Technology	Rabbit
mTOR	ab2732	Abcam	Rabbit

### 2.6 Co‐immunoprecipitation

HEL cells (15 million for each condition) were rinsed with cold PBS and lysed on ice‐cold CHAPS buffer lacking NaCl (40 mmol/L HEPES [pH 7.4], 0.3% CHAPS, protease and phosphatase inhibitors) to isolate mTORC1 complex.^17^ Cell debris was removed from the lysates by centrifugation at 16 200*g* for 15 minutes at 4°C, followed by pre‐cleaning with Protein A/G PLUS‐Agarose (sc‐2003, Santa Cruz Biotechnology). mTOR antibody (Table 1) for co‐immunoprecipitation was added to the lysate (1 mg/mL) and incubated ON at 4°C. Protein A/G (20 μL) were added to the antibody and lysate mixture and incubated 1 hour at 4°C in a rotator. The mock control (beads and whole cell lysates without adding antibody) was used to exclude the false interaction of lysate proteins with the beads. Immunoprecipitates and mock controls were washed once with CHAPS buffer lacking NaCl and three times with CHAPS buffer containing 150 mmol/L NaCl. Samples were eluted in 5× Laemmli buffer at 95°C for 10 minutes and resolved on 6% SDS‐PAGE as described above. mTOR‐Raptor complex was evaluated using mTOR and Raptor antibodies (Table 1) as previously described.

### 2.7 RNA extraction and qRT‐PCR analysis

Total RNA was extracted using TRIzol Reagent (Ambion, Thermo Fisher Scientific), following the manufacturer's instructions. 1 µg on total RNA was used as a template for the reverse transcription reaction. Expression levels of PIM1 (Hs_00171473_m1), PIM2 (Hs_00179139_m1), PIM3 (Hs00420511_g1), CD177 (Hs00360669_m1), Socs‐1 (Hs00705164_s1) and Socs‐3 (Hs00269575_s1) were evaluated with TaqMan technology (TaqMan Universal Master Mix, Thermo Fisher Scientific) with C1000 Thermal Cycler CFX96 Real‐Time System (Bio‐Rad). qRT‐PCR data were analysed by Bio‐Rad CFX Manager 3.1 software (Bio‐Rad). Genes expression was normalized respect to ABL (Hs00245445_m1).

### 2.8 Statistical analysis

Statistical analyses were performed using the two‐tailed Mann‐Whitney *U* test and paired *t* test. All the analysis with confidence level major of 95% are indicated like significant and marked as followed: *P ≤ 0.05; **P ≤ 0.01; ***P ≤ 0.001.

## 3 RESULTS

### 3.1 Curcumin induces apoptosis and inhibits viability in HEL cells

**Figure 1:**
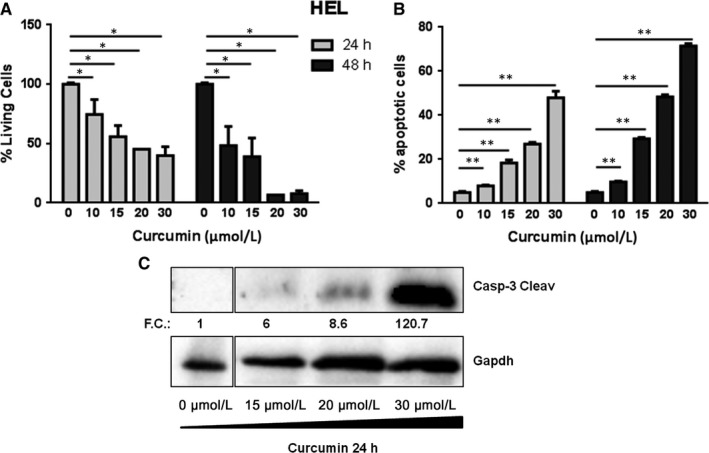
HEL cells treated with different concentration of curcumin (0‐30 µmol/L) for 24 and 48 h. A, Effect of curcumin on cell viability. The percent of live cells was calculated by normalizing with the control (n = 5). B, Effect of curcumin on apoptosis (n = 5). C, Caspase‐3 Cleaved expression was evaluated by SDS‐PAGE. The GAPDH level was used as loading control. The relative intensity of each band is shown under the blot as fold change (FC) compared to untreated control, to which a value of 1 unit was assigned. Statistical analyses were performed using the two‐tailed Mann‐Whitney *U* test, comparing conditions two by two respect to not treated condition. All the analysis with confidence level major of 95% are indicated like significant and marked as followed: *P ≤ 0.05; **P ≤ 0.01;

To explore the effects of curcumin on cell death and viability, HEL cells were treated with various concentrations of curcumin (0‐30 µmol/L) for 24/48 hours, proliferation and apoptosis were evaluated by FACS and immunoblotting. The viability of the HEL cells exposed to curcumin was significantly lower compared to control cells even at low concentrations. In addition, the curcumin growth inhibitory effect was dose and time‐dependent, reaching the maximum effect at the dose of 20 µmol/L for 48 hours, by reducing the proliferation of 93% (Figure 1A). The results obtained evaluating apoptosis with Annexin V integrate and confirm viability analysis. In particular, we showed that the percentage of apoptotic cells increase of 2.1, 3.6, 5.0 and 5.9 times at 24 hours and 2.1, 4.7, 6.3 and 8.6 at 48 hours, respectively after treatment with 10, 15, 20, and 30 µmol/L of curcumin (Figure 1B). Furthermore, to investigate the intrinsic apoptosis at the protein level, treated HEL cells were harvested at 24 hours and analysed by Western blot. As shown in Figure 1C, the protein expression of cleaved caspase‐3 was markedly increased in the curcumin‐treated HEL cells according to drug concentration. In particular, a curcumin concentration of 30 µmol/L showed a sharp increase of cleaved caspase‐3. This data confirmed the FACS analysis and indicated that curcumin induced apoptosis in a dose‐dependent manner in JAK2 V617F‐mutated cell line.

### 3.2 Curcumin affects JAK2/STAT pathway in HEL cell line

**Figure 2:**
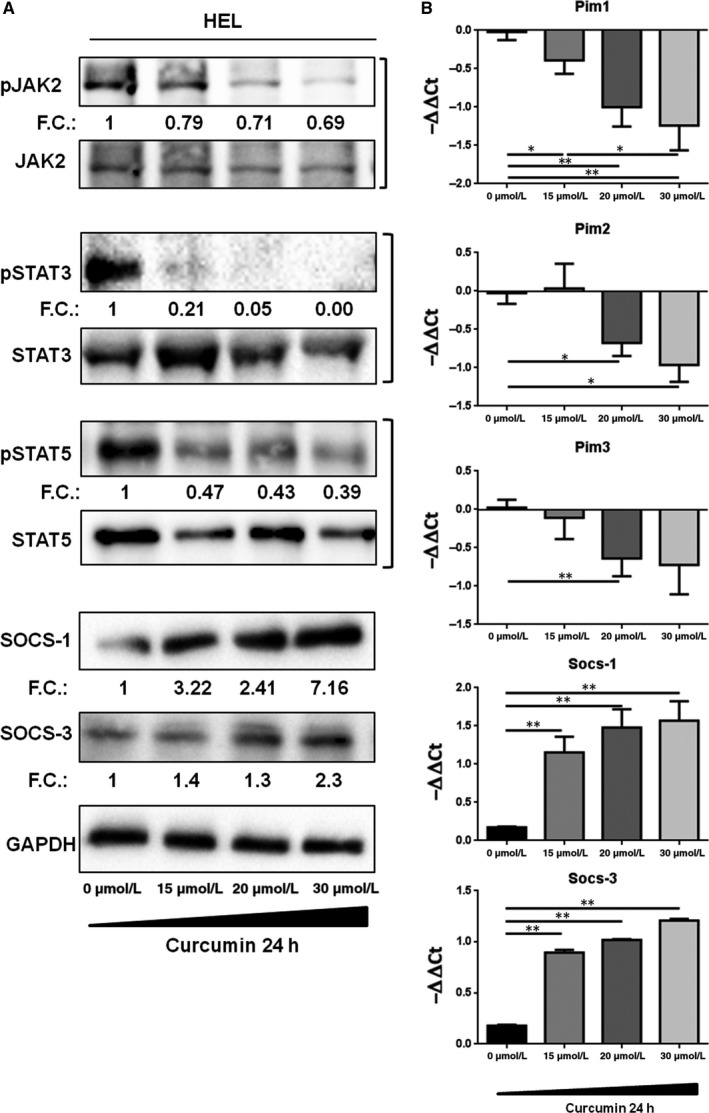
HEL cells treated with different concentration of curcumin (0‐30 µmol/L) for 24 h. A, The indicated proteins of JAK2/STAT5 signalling were detected by SDS‐PAGE. Representative Western blots were shown (n = 3). The relative intensity of each band is shown under each blot as fold change (FC) compared to untreated control. B, The mRNA expression levels of PIM family members, Socs‐1 and Socs‐3 were evaluated by qRT‐PCR. Results are expressed as −∆∆Ct (n = 7). Statistical analyses were performed using the two‐tailed Mann‐Whitney *U*test, comparing conditions two by two respect to not treated condition. All the analysis with confidence level major of 95% are indicated like significant and marked as followed: *P ≤ 0.05; **P ≤ 0.01;

To investigate the modulation of JAK/STAT pathway, HEL cells were treated with various concentrations of curcumin (0‐30 µmol/L) for 24 hrs and JAK2, STAT3 and STAT5 phosphorylation status was evaluated by immunoblotting. Data showed that curcumin reduced the tyrosine phosphorylation of JAK2, STAT3 and STAT5 in a dose‐dependent manner. Although the cells treated with curcumin 20 and 30 µmol/L showed a reduction of JAK2 phosphorylation of about 30%, the functionality of JAK2 to phosphorylate its effectors was strong compromised. The phosphorylation of STAT5 and STAT3, the direct effectors of JAK2, was strongly inhibited by curcumin. Indeed, we observed that phosphorylation of STAT5 was 60% reduced, while STAT3 was extremely sensitive to curcumin‐mediated JAK2 inhibition and its phosphorylation decreased to 20% even at the lower curcumin dose and was completely blocked at higher concentrations. Furthermore, the protein expression of SOCS‐1 and SOCS‐3, negative regulators of JAK/STAT pathway, resulted clearly up‐regulated after curcumin treatment. Indeed, their expression increased of three and 1.4 times, respectively, even at the lower curcumin dose and of seven and two times, respectively, at higher concentrations (Figure 2A). A similar behaviour was observed also at mRNA level; indeed, curcumin significantly increased the expression of SOCS‐1 and SOCS‐3, displaying an up‐regulation of about six times even at 15 µmol/L of curcumin (*P* < 0,01) (Figure 2B). These results were further corroborated by measuring the transcriptional activity of JAK downstream effectors STATs on mRNA expression of PIM family members. Curcumin significantly decreased the expression of PIM1, PIM2 and PIM3, showing a down‐modulation of about two times compared to control when cells were treated with a concentration higher than 20 µmol/L (PIM1 and 3 *P* < 0.01 and PIM2 *P* < 0.05) (Figure 2B).

### 3.3 Curcumin modulates mTORC1 signalling by the inhibition of PDK and AKT in HEL cells line

**Figure 3:**
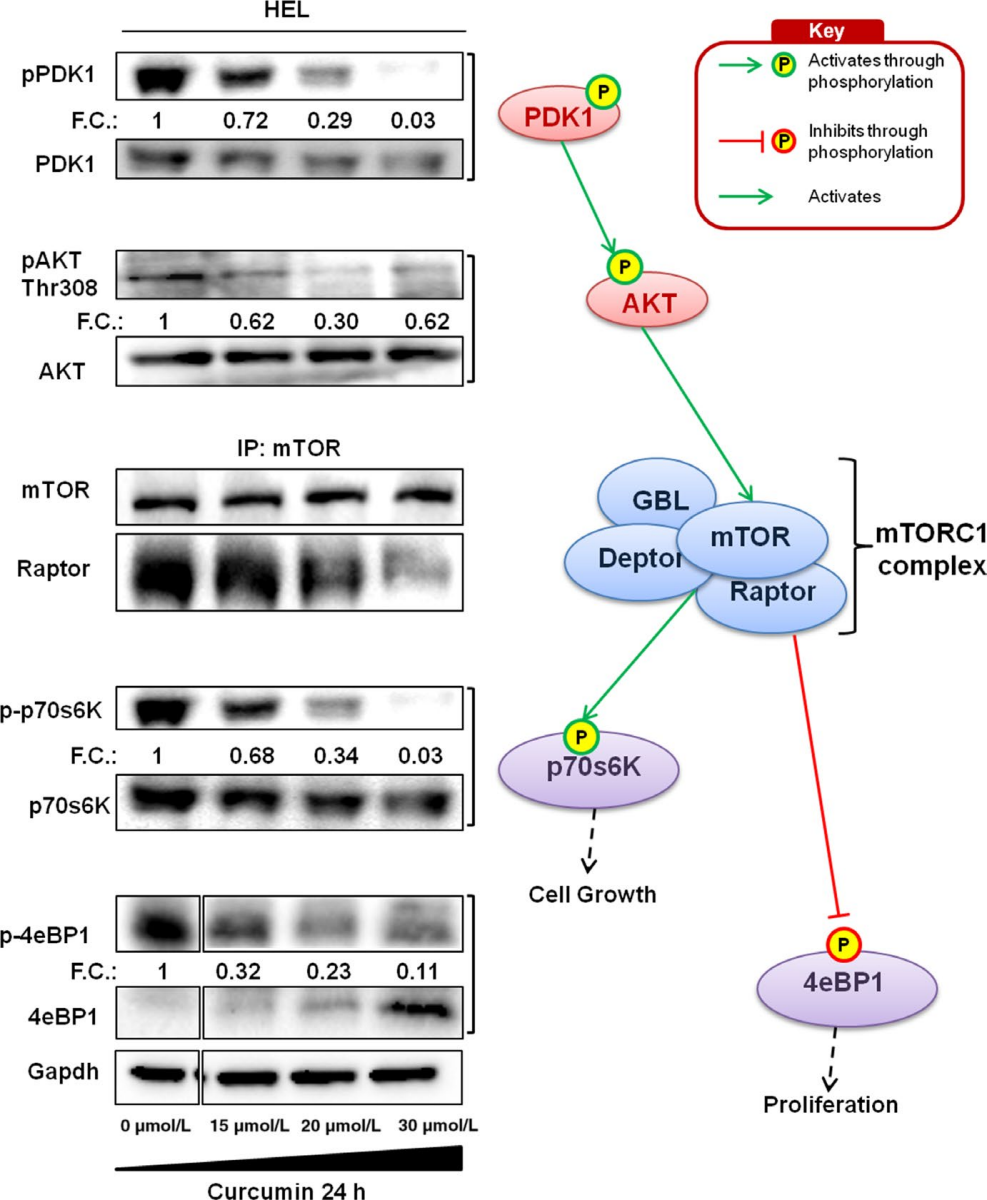
HEL cells treated with different concentrations of curcumin (0‐30 µmol/L) for 24 h. Phosphorylation status of PDK1 and AKT (n = 4), co‐immunoprecipitation of mTOR‐Raptor complex (n = 3) and phosphorylation status of p70s6k and 4EBP1 (n = 4) were evaluated by SDS‐PAGE. The relative intensity of each band is shown under each blot as the fold change (FC) compared to untreated control. The analysed pathways were indicated on right side

To investigate how curcumin affects mTORC1 pathway, HEL cells were treated with various concentrations of curcumin (0‐30 µmol/L) for 24 hours and analysed by immunoblotting. Our results indicated that curcumin affects the principal modulator of mTORC1: AKT. Phosphorylation of AKT in Thr308 was reduced in dose‐dependent manner and its principal activator PDK was inhibited by low dose curcumin and it appeared unphosphorylated at the maximum concentration utilized (Figure 3). The consequence was the reduction of the complex between mTOR and Raptor, as observed in Figure 3, especially at the highest drug concentration. Furthermore, by analysing the signal cascade, we observed the deregulation of the principal downstream effectors of mTORC1. In particular, we showed the dephosphorylation of p70s6K, which is usually activated by phosphorylation, and the increase of unphosphorylated form of 4eBP1, usually inhibited through mTORC1 phosphorylation.

### 3.4 Curcumin induces apoptosis and reduces PIM family members and CD177 expression in JAK2 mutated MPNs patients

**Figure 4:**
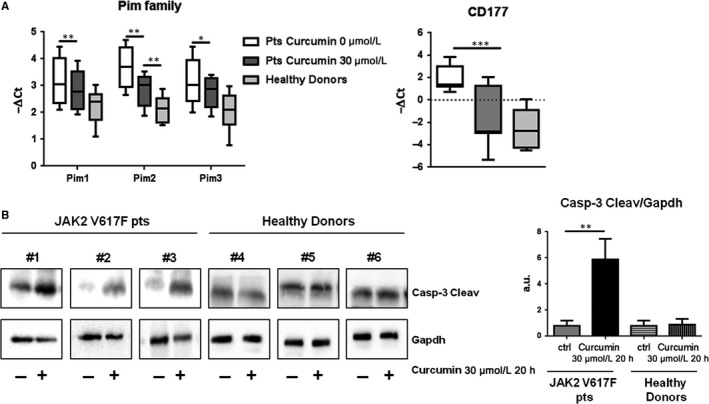
Leucocytes of MPNs patients (pts) were treated with 30 µmol/L curcumin for 20 h. A, The mRNA expression of PIM family members and CD177 was evaluated by qRT‐PCR in MPNs pts and healthy controls. Results are expressed as −∆Ct (n = 20). Statistical analyses were performed using paired *t* test to compare treated and not treated MPNs patients' cells and the two‐tailed Mann‐Whitney *U*test to compare treated patients with healthy donors' cells. All the analysis with confidence level major of 95% are indicated like significant and marked as followed: *P ≤ 0.05; **P ≤ 0.01; ***P ≤ 0.001. B, Caspase‐3 Cleaved expression was evaluated by SDS‐PAGE. The GAPDH level was used to normalize data. Representative Western blots of three patients and three healthy controls were shown

Due to the promising results in vitro, we tried to evaluate the effect of curcumin also in patient specimens. For these purposes, leucocytes from JAK2 mutated patients were treated with curcumin at the highest concentration utilized in vitro (30 µmol/L) for 20 hours and the expression of PIM family members and CD177 were evaluated by qRT‐PCR. The results were in accordance with in vitro analysis and showed that PIM1, PIM2, PIM3 and CD177 mRNA expression was significantly down‐regulated in cells treated with curcumin compared to not treated samples (PIM1 *P* = 0.0014; PIM2 *P* = 0.0073; PIM3 *P* = 0.011; CD177 *P* < 0.0001) (Figure 4A). Interestingly, except for PIM2 (*P* = 0.025) (Figure 4A), the mRNA expression of PIM family members and CD177 after treated return to the levels of healthy subjects (PIM1 *P* = 0.1; PIM3 *P* = 0.07 and CD177 *P* = 0.58). Furthermore, the expression of cleaved caspase‐3, evaluated by SDS‐PAGE, was significantly up‐regulated in patients' cells treated with curcumin (*P* < 0.001), while it has not changed in cells from healthy donors (Figure 4B). These results suggest that curcumin treatment affects JAK/STAT pathway and induces apoptosis in cells from *JAK2 V617F* mutated patients.

## 4 DISCUSSION

The majority of Philadelphia negative MPNs shows the single base *JAK2 V617F* mutation that results in a constitutive activation of the JAK/STAT pathway, conferring a proliferative advantage and apoptosis inhibitions. The discovery of *JAK2* mutations allowed the development of JAK2 inhibitors for target therapies, including ruxolitinib, approved for patients with primary or secondary myelofibrosis and hydroxyurea‐resistant or intolerant PV patients. Although ruxolitinib has brought enormous benefits to patients, evidence that JAK2 inhibition can cure the disease, at least in PMF, remains debated.^18^, ^19^ The difficulty in treating patients affected by MPNs encourages the identification of new effective drugs, including some natural compounds, like curcumin. Curcumin has been found to exert its anti‐inflammatory and anti‐carcinogenic effects by targeting JAK2 signalling in different type of neoplasms and injuries,^14^, ^20^ but the effects of this phytochemical on JAK2‐mutated cells have been poorly studied until now.

In this work, we investigated, at both RNA and protein levels, the effects of curcumin on JAK2/STAT pathway, known to be altered in MPNs patients. Our data are in accordance to what has been already published by Chen and colleagues, who demonstrated that curcumin up‐regulate SOCS‐1 and SOCS‐3 which are important negative regulators of JAK2.^15^ In addition, we showed that curcumin strongly inhibited the proliferation and induced apoptosis in a dose and time‐dependent manner in HEL cells. Furthermore, we found that curcumin markedly reduced JAK2 phosphorylation and, consequently, the activation of its downstream effectors STAT3 and STAT5 in HEL cells, according to the results obtained by Zhu et al in severe acute pancreatitis^14^ and by Kim et al in rat primary microglia.^20^ The inhibition of JAK2/STAT pathway, observed in our study, induced the down‐regulation of PIM family members, PIM1, PIM2 and PIM3 in HEL cell line and in patient specimens. These small kinases are known to be involved in leukemogenesis and in ruxolitinib resistance in MPNs cells.^21^, ^22^ As Hitosugi et al identified that constitutively activated JAK2 regulates AKT through the phosphorylation of PDK1,^23^ we focused our attention on this pathway. Our data showed that curcumin‐mediated inhibition of JAK2 reduces the activation of PDK1 and, consequently, the Thr‐308 AKT phosphorylation in HEL cells. It has been reported that AKT directly regulates mTORC1 signalling,^24^ so we investigated the effect of curcumin on this pathway. In agreement with the results obtained by Beevers et al in human rhabdomyosarcoma cells, our results in HEL cells showed that curcumin negatively regulated the mTORC1 complex formation. As a consequence, we observed a strong inhibition of mTORC1 downstream effectors 4eBP1 and p70s6K phosphorylation, both involved in cell growth and proliferation.^16^ This data are in accordance with the hypothesis that mTORC1 is a JAK2 downstream pathway and it is involved in MPNs pathogenesis.^25^ Due to the promising data obtained in cell line, we tried to evaluate the effect of curcumin also in patient samples. Our preliminary data showed that the in vitro curcumin treatment of cells from MPNs patients significantly reduced the expression of PIM family members, bringing back their mRNA expressions to comparable levels with healthy subjects ones. The curcumin effect was strengthened by the observation that CD177, an antigen over expressed in neutrophils of the majority of MPNs patients, was down‐regulated by curcumin.^26^

Ishida et al demonstrated that HEL cells are only partially dependent on JAK2 V617F for survival and this may explain the very limited effect of ruxolitinib and its inefficiency to eradicate JAK2‐mutated clone in MPNs patients.^27^ Interestingly, in this study, we observed that curcumin affects both proliferation and survival of HEL cells, suggesting that its role in MPNs could be more effective in blocking neoplastic cells respect to the common JAK2 inhibitors, such as ruxolitinib. In vitro studies support this hypothesis by indicating that co‐treatment of mTOR and JAK2 inhibitors resulted in synergistic activity against JAK2 V617F‐mutated cells,^5^ although mTORC1 inhibitors can cause numerous side effects that could prevent their use.^28^ In contrast, curcumin turned out to be safe and non‐toxic in many different trials,^29^, ^30^ so its use for MPNs treatment could represent an excellent alternative to common mTORC1 inhibitors. Although the curcumin employment for MPNs treatment needs to be further investigated, our preclinical data encourage its use for the future therapies.

In conclusion, this study showed that curcumin exerts an antitumor effect on human JAK2‐mutated cells by inducing apoptosis and inhibition of proliferation, through the regulation of both JAK2/STAT and mTORC1 pathways. These findings suggest that curcumin seems to be a promising nutraceutical compound that should be further evaluated in different pharmaceutical formulation for the treatment of MPNs.

## CONFLICT OF INTEREST

The authors declare that they have no competing interests.

## AUTHOR CONTRIBUTIONS

JP and VR designed the study, performed the experiments and wrote the manuscript. ML analysed data and revised the manuscript. CP performed FACS analysis. CC, ES, LP and AC collected samples. EB and BP supervised the experiments. TB provided samples. CF and DC supervised the experiments and wrote the manuscript. All authors read and approved the final manuscript.
